# The impact of Covid-19-related distress on general health, oral behaviour, psychosocial features, disability and pain intensity in a cohort of Italian patients with temporomandibular disorders

**DOI:** 10.1371/journal.pone.0245999

**Published:** 2021-02-02

**Authors:** Giacomo Asquini, Andrea Edoardo Bianchi, Giulia Borromeo, Matteo Locatelli, Deborah Falla

**Affiliations:** 1 Centre of Precision Rehabilitation for Spinal Pain (CPR Spine), School of Sport, Exercise and Rehabilitation Sciences, College of Life and Environmental Sciences, University of Birmingham, Birmingham, United Kingdom; 2 Craniomandibular Physiotherapy Service, Italian Stomatologic Institute, Milan, Italy; 3 IRCCS San Raffaele Scientific Institute, Milan, Italy; Universiteit Antwerpen, BELGIUM

## Abstract

This study aimed to understand the impact of COVID-19 distress on psychological status, features of central sensitization and facial pain severity in people with temporomandibular disorders (TMDs). In this prospective cohort study, 45 adults (19 chronic, 26 acute/subacute TMD) were recruited prior to the COVID-19 outbreak. Baseline assessment took place before the outbreak while a follow-up was performed immediately after the lockdown period. Multiple variables were investigated including age, gender, perceived life quality, sleep quality, anxiety and depression, coping strategies, central sensitization, pain intensity, pain-related disability and oral behaviour. COVID Stress Scales (CSS) were applied at follow-up to measure the extent of COVID-related distress. CSS were significantly higher in those with chronic TMDs compared to those with acute/subacute TMDs (p<0.05). In people with chronic TMD, the variation in anxiety and depression from baseline to follow-up was significantly correlated with scores on the CSS (r = 0.72; p = 0.002). Variations of the central sensitization inventory (r = 0.57; p = 0.020) and graded chronic pain scale (r = 0.59; p = 0.017) were significantly correlated with scores on the CSS. These initial findings indicate that people with chronic TMD were more susceptible to COVID-19 distress with deterioration of psychological status, worsening features of central sensitization and increased chronic facial pain severity. These findings reinforce the role of stress as a possible amplifier of central sensitization, anxiety, depression, chronic pain and pain-related disability in people with TMDs.

**Trial Registration**: **ClinicalTrials.gov ID:** NCT03990662.

## Introduction

The worldwide spread of the new zoonotic virus SARS-COV2 led the World Health Organization (WHO) to declare the COVID-19 pandemic on the 11th of March 2020 [1]. At the beginning of June 2020, more than 7 million people were affected by SARS-COV2 with more than 400000 deaths [2]. Italy took country-level measures on March 9^th^ 2020, impacting on everyday life with unprecedented restrictions [3].

The lockdown along with the fear of contracting and transmitting the virus resulted in panic, anxiety, obsessive behaviours, depression and even post-traumatic stress disorder (PTSD) [4, 5]. Psychological distress, PTSD and depression were found after SARS-COV2 quarantine restrictions, and isolation-related anxiety symptoms were also reported during and after the Middle East Respiratory Syndrome epidemic [6–8]. The psychosocial impact of COVID-19 can impact on musculoskeletal pain, in particular for stress-related pain conditions such as temporomandibular disorders (TMDs) [9–12].

Several studies have investigated the relationship between stress and TMDs, providing evidence that psychological distress is associated with high levels of TMD pain and pain-related disability [13, 14]. Case-control studies observed that stress, anxiety, depression and catastrophizing scores were significantly higher in people with TMD than in an asymptomatic population [15, 16]. Moreover, people with orofacial pain have reported that stress contributed to the onset, development and maintenance of their pain [17–19].

Previous studies examined the impact of global stressor events (e.g. war) on people with TMD revealing a tremendous influence on TMD signs and symptoms [20–22]. With regards to the impact of COVID-19 on patients with TMDs, two cross-sectional, case-control studies have recently been reported but, given the nature of the research design, these studies were not able to examine the variation of TMD signs and symptoms pre and post COVID-19 lockdown in relation to the psychosocial distress experienced [23, 24]. Additionally, previous reports did not differentiate the analysis between chronic and acute/subacute forms and were not able to examine several key variables in people with TMDs such as general health variables, oral behaviours, coping strategies and disability.

This cohort study aims to uniquely investigate the impact of COVID-19-related distress on general health variables, oral behaviours, psychosocial features, disability and pain intensity in a group of 45 Italian patients with TMD. Such findings will inform future research on the association between stress and TMDs and pain-related behaviour changes in people with TMDs due to global stressor events.

## Materials and methods

This study reports findings from a prospective cohort study investigating predictors associated with pain reduction in patients with TMD following manual therapy [25]. The study took place at the TMJ-Unit of the Italian Stomatological Institute (Dental Hospital) in Milan, Italy. All participants were informed about all aspects of the research and provided written consent. Ethical approval was obtained from the Ethics Committee of the Fondazione IRCCS Ca’ Granda Ospedale Maggiore Policlinico (acceptance no. “534_2019bis”) and the study was conducted in accordance with the Declaration of Helsinki.

### Participants, recruitment and procedure

From July 2019 to February 2020, 45 patients (19 with chronic TMD and 26 with acute/subacute TMD) were recruited from the TMJ Unit of the Italian Stomatological Institute (Dental Hospital) in Milan, Italy. One dentist with expertise in TMD assessment and management (more than 10 years), screened patients and verified the TMD diagnosis according to the Diagnostic Criteria for Temporomandibular Disorders (DC/TMD) following the Italian protocol [26, 27]. Patients were included if they were adults (≥18 years) with one or multiple TMD diagnoses according to DC/TMD and had not received therapeutic interventions for their TMD in the past six months [26]. They were required to be able to use and understand written and verbal Italian language and provide written informed consent. Patients were excluded if they presented with TMD pain related to rheumatoid/inflammatory arthritis, or demonstrated any physical/mental status that could affect the study results.

After inclusion, participants were assigned to a single physiotherapist with more than 5 years of experience in TMD assessment and treatment for baseline data collection. Subsequently, participants received their therapy for their TMD consisting of manual therapy for the first month followed by exercises and an oral appliance if needed (for full details see Asquini et al. 2020) [25]. When the pandemic started, all participants had already completed baseline data collection and at least one month of therapy. From March 2020 to May 2020, all participants were affected by Italian measures to limit the spread of COVID-19. These measures gradually were removed in June 2020 in accordance with the reduction of the number of infected people in Italy. Follow-up data collection was made by the same baseline assessor and took place in June 2020. Each participant was called (maximum 3 attempts) to confirm an appointment to complete the follow-up measures.

### Observed variables and outcome measures

Observed variables were selected according to previous research on putative risk factors for TMDs and abnormal musculoskeletal pain processing [28, 29].

#### Demographical variables

The participants age and gender were recorded.

#### General health variables

*Visual Analogue Scale—Quality of Life (VAS-QoL)*. Health-related quality of life was evaluated through a visual analogue scale (range 0–100) representing ‘worst’ to ‘best’ possible health [30, 31].

*Sleep Quality*. An 11-point Numerical Rating Scale [NRS] was adopted to estimate sleep quality, where 0 is ‘best possible sleep’ and 10 is ‘worst possible sleep’ [32]. This scale was used to assess the average sleep quality related to the preceding 6-months at baseline and related to the lockdown period at the follow-up [33].

#### Psychosocial features

*The Hospital Anxiety and Depression Scales (HADS)*. Depression and anxiety were measured with the Italian version of the HADS at baseline and at the follow-up assessment [34, 35]. HADS is made up of two 7 items subscales [anxiety: HADS-A; depression: HADS-D]. The score ranges from 0 to 21 with higher values indicating greater levels of anxiety and depression [36]. HADS has excellent concurrent validity in comparison to other depression/anxiety scales, and adequate to excellent internal consistency (HADS-A [0.68–0.93]; HADS-D [0.67–0.90]) [36].

*Coping Strategies Questionnaire 27 (CSQ-27)*. The Italian version of the CSQ-27 was applied to assess strategies for coping with pain at baseline and at the follow-up [37]. This questionnaire includes six domains: Distraction, Catastrophizing, Ignoring pain sensations, Distancing from pain, Coping self-statements, and Praying. Participants were asked to score the specific strategies utilising a seven-point Likert scale for each domain. Possible scores range from 0 “never do that” to 6 “aways do that” [38]. The catastrophizing domain score (CSQ-CAT) was unbundled from other scores since it clustered with pain-related distress items, unlike other domains [39]. Acceptable internal consistency [Cronbach’s alpha estimates ranging from 0.72 to 0.86] and satisfactory construct validity has been reported [38].

#### Pain and TMD characteristics

The duration of pain (measured in days) was obtained at baseline from open hospital records and the patient interview. According to previous definitions, participants reported pain for more than 6 months were considered as chronic TMD [40, 41]. By contrast, participants with a history of pain duration of less than 6 months were considered non-chronic TMD.

*Central Sensitization Inventory (CSI)*. Participants completed the part A of the Italian version of the CSI at baseline and follow-up [42]. This questionnaire is composed of 25 items with five possible answers ranging from ‘never’ (0) to ‘always’ (4) concerning current health symptoms. Significant test-retest reliability and internal consistency were found for CSI in people with and without pain [43]. Cronbach’s alpha of the Italian versions of the CSI is 0.87 [42].

*Characteristic pain intensity and disability*. The Italian version of the Research Diagnostic Criteria for TMD (RDC/TMD) questionnaire Axis II Graded Chronic Pain Scale (GCPS) version -2.0 [www.rdc-tmdinternational.org] was administered by following the DC/TMD protocol recommendations [9, 26, 44–47]. A Cronbach's alpha of 0.84 demonstrated good internal consistency in TMDs [48]. In this study, the GCPS was used to appraise facial pain severity related to the preceding 6-months at baseline and related to the 3 months of the lockdown period at follow-up. This scale unifies pain intensity and pain-related disability into one of the five ordinal categories of chronic facial pain severity [49]. Pain intensity is measured through the characteristic pain intensity (CPI) score (range: 0–100) [47–49]. A disability score (range: 0–6) is obtained from a combination of the number of disability days and the disability level [47–49].

*Oral behaviour*. The Italian version of the RDC/TMD questionnaire Axis II Oral Behaviours Checklist (OBC) [www.rdc-tmdinternational.org] was administered at baseline and at the follow-up by following the DC/TMD protocol recommendations [9, 26, 50]. The OBC is a 21 item questionnaire with five possible answers ranging from ‘none of the time’ (0) to ‘all of the time’ (4), referring to activities related to the preceding month involving the jaw such as clenching or grinding the teeth. This scale has good psychometric properties and validity [50–52].

#### COVID Stress Scales (CSS)

The CSS were used to assess COVID-19-related distress at follow-up [53]. These scales were developed and initially validated in a representative population of Canada and the United States. An Italian translation of the CSS is reported as S1 Appendix. The CSS are a 36-items scales composed of five domains: danger and contamination fears, fears about economic consequences, xenophobia, compulsive checking and reassurance-seeking, and traumatic stress symptoms about COVID-19. All items are scored on a 5-point scale ranging from 0 to 4, with elevated values indicating high COVID-related impact. Current evidence revealed that CSS worked with acceptable values of reliability and validity [53].

### Potential bias

All observed variables and outcomes measures were evaluated at baseline and follow-up by the same independent assessor to minimise detection bias. The number and reason for exclusion of participants throughout the life course of the study is reported limiting attrition bias.

### Statistical analysis

The study sample size was not calculated a-priori because of the unpredictability of the COVID-19 pandemic. Data analysis was performed using IBM SPSS (version 22). A Kolmogorov-Smirnov test confirmed non-normal distributions of the data. Therefore, descriptive statistics were calculated (median, first and third quartile) for each variable at baseline and the follow-up assessment. A Mann Whitney test was used to compare the COVID related distress between non-chronic and chronic TMD participants. A Spearman test was used to correlate the variations from baseline to follow up in general health variables, psychosocial features and TMD characteristics with the extent of COVID related distress. The statistical significance level was set at p ≤ .05. All missing data are reported. The participants with missing data at baseline or follow-up were excluded from the statistical analysis.

## Results

The number of participants examined for eligibility was 73; 28 were excluded because they did not meet the inclusion/exclusion criteria thus 45 were confirmed as eligible and were included in the study. According to the taxonomy of the DC/TMD, 10 participants presented with a temporomandibular joint disorder, 12 a masticatory muscle disorders and 23 a mixed disorder [45]. Forty participants completed the follow-up, while 5 participants were uncontactable and therefore did not complete the follow-up assessment.

### Demographic characteristics

The final analysis was conducted on data from 40 participants with complete baseline and follow-up data. The age was similar in acute/subacute (median = 29 years) and chronic TMD participants (median = 28 years). The gender distribution was also similar between those with acute/subacute TMD (88% females) and chronic TMD (94% females).

### General health variables, psychosocial features and TMD characteristics

General health variables, psychosocial features and TMD characteristics are reported in Table 1. The duration of pain in those with chronic pain (615 days) was much greater than in acute/subacute participants (60 days), as expected. The other characteristics at baseline were similar between the two groups, except for a slightly higher CSI score in those with chronic TMD (median = 40.50) compared to acute/subacute TMD (median = 27.00). In the acute/subacute group, variations from baseline to follow up showed slight improvement in quality of life, sleep quality, coping strategies, central sensitization, pain intensity, disability and oral behaviour. By contrast, in those with chronic TMD, only sleep quality improved slightly from baseline to follow up, while quality of life, coping strategies, central sensitization and oral behaviour worsened. Anxiety and depression worsened from baseline to follow up in both groups albeit more so for those with chronic TMD.

**Table 1:** Median, first and third quartile of general health variables, psychosocial features and TMD characteristics at baseline and the follow-up.

	Acute/subacute TMD (n = 24)			Chronic TMD (n = 16)		
	Baseline	Follow up	Change from baseline to follow up	Baseline	Follow up	Change from baseline to follow up
Pain duration (days)	60.00 (26.25; 90.00)	-	-	615.00 (363.75; 1186.25)	-	-
VAS- QoL	80.00 (70.00; 86.25)	82.50 (80.00; 90.00)	3.50 (-5.00; 10.00)	72.50 (53.75; 86.25)	70.00 (55.00; 80.00)	-6.50 (-15.00; 0.00)
NRS Sleep Quality	5.00 (2.00; 7.00)	5.00 (4.00; 6.25)	1.00 (-2.00; 2.00)	5.00 (3.00; 6.25)	7.00 (4.75; 8.00)	1.50 (-0.25; 3.00)
HADS	8.00 (6.00; 14.50)	11.00 (7.75; 16.00)	3.00 (-3.25; 7.00)	12.50 (8.75; 20.50)	18.50 (9.00; 27.25)	5.00 (2.50; 7.25)
CSQ	45.50 (38.50; 72.25)	54.50 (45.25; 70.00)	0.50 (-6.75; 17.25)	45.50 (30.00; 56.50)	46.50 (33.75; 56.25)	-3.00 (-13.25; 9.00)
CSQ- CAT	9.00 (3.00; 14.00)	6.50 (1.75; 10.75)	-2.00 (-5.25; 3.50)	11.50 (2.75; 18.25)	12.00 (2.75; 24.50)	4.00 (0.75; 6.00)
CSI	27.00 (19.75; 32.25)	27.00 (20.25; 32.25)	-1.00 (-5.00; 5.25)	40.50 (34.75; 47.00)	44.00 (24.75; 55.00)	3.00 (-7.00; 10.00)
GCPS	2.00 (1.00; 3.25)	1.00 (1.00; 1.00)	-1.00 (-2.00; 0.00)	2.00 (1.00; 3.00)	2.50 (1.00; 4.00)	0.00 (0.00; 1.00)
OBC	33.00 (28.25; 38.50)	26.00 (18.50; 33.25)	-7.00 (-13.00; 0.00)	28.50 (22.75; 38.25)	41.50 (30.75; 44.25)	7.50 (-5.25; 19.25)

### COVID related distress

The COVID related distress measured with the CSS was significantly higher for those with chronic TMD (median = 52.50) compared to those with acute/subacute TMD (median = 30.00, P = .027).

### Correlation between CSS and variations in clinical outcome measures over the lockdown period

**Fig 1:**
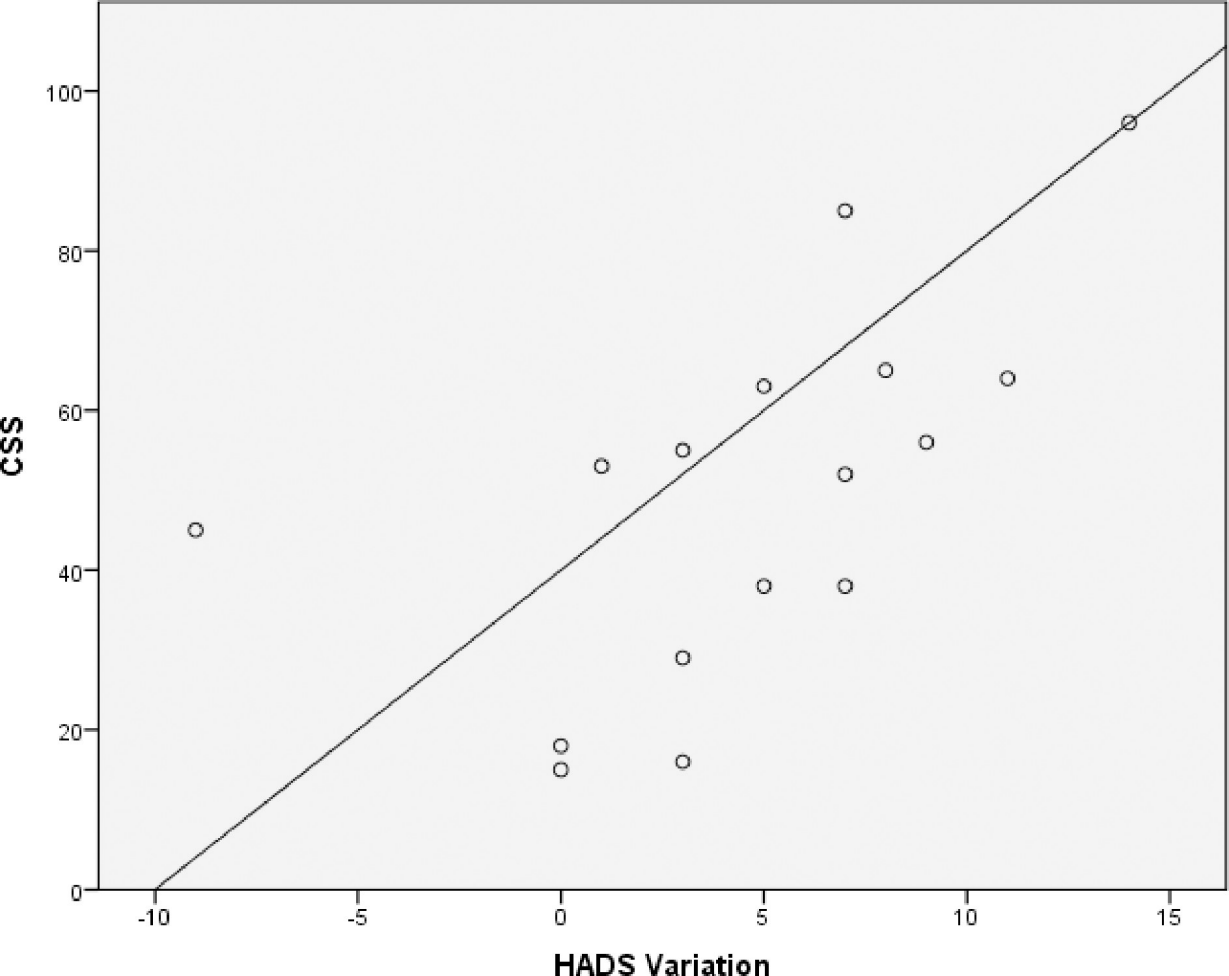
Correlation between CSS and HADS variation in those with chronic TMD. In participants with chronic TMD, HADS variation from baseline to follow up was significantly correlated with CSS (r = 0.72, p = .002).

No statistically significant correlations were found between the CSS score and the change in general health variables, psychosocial features, TMD characteristics in those with acute/subacute TMD. For the participants with chronic TMD, HADS variation from baseline to follow up was significantly correlated with CSS (r = 0.72, p = .002) (Fig 1). Variation of the CSI (r = 0.57, p = .020) and GCPS (r = 0.59, p = .017) were also significantly correlated with CSS. No other correlations were significant (Table 2).

**Table 2:** Correlations between the CSS score and the change in general health variables, psychosocial features and TMD characteristics.

	CSS acute/subacute TMD (n = 24)		CSS chronic TMD (n = 16)	
	Correlation coefficient	Significance	Correlation coefficient	Significance
ΔVAS-QoL	0.21	p = .321	-0.47	p = .069
ΔNRS Sleep Quality	-0.21	p = .314	0.27	p = .312
ΔHADS	0.10	p = .630	0.72	p = .002^a^
ΔCSQ	0.16	p = .461	-0.49	p = .052
ΔCSQ-CAT	0.05	p = .815	0.49	p = .052
ΔCSI	-0.03	p = .879	0.57	p = .020^a^
ΔGCPS	-0.08	p = .693	0.59	p = 0.017^a^
ΔOBC	-0.29	p = .172	0.30	p = .261

Δ = change of the variable from baseline to follow-up

^a^ indicates statistically significant result, p<0.05.

## Discussion

This study examined the impact of COVID-19-related distress on general health variables, oral behaviours, psychosocial features, disability and pain intensity in a cohort of Italian patients with either chronic or acute/subacute TMD. The CSS revealed significantly different levels of COVID-19 related stress experienced during lockdown in these two groups; those with chronic TMD were significantly more influenced by the fear of contracting and transmitting the virus with the consequent behaviour of compulsive checking, reassurance-seeking and traumatic stress symptoms. These findings support previous research investigating stress-related behaviour and chronic TMD [14, 54, 55]. As revealed in this study and earlier studies, a global stressor event (e.g. COVID-19 pandemic, war) can strongly affect the life of patients with TMDs [20–24]. Our results revealed that participants who presented with chronic TMD before the COVID-19 pandemic were more susceptible to COVID-related stress in comparison to those with acute/subacute TMD.

Previous research has revealed changes in perceived quality of life, sleep quality, anxiety and depression, coping strategies, global facial pain severity and oral behaviour in people with TMD [18, 41, 56–58]. Such studies however have investigated these variables in people with TMD versus asymptomatic individuals but no study had prospectively examined the variation of these variables in relation to the psychosocial distress experienced during a global stressor event [18, 41, 56–58]. Previous cross-sectional, case-control studies on the influence of COVID-19 on TMDs showed a significantly higher impact of COVID-related stress in those with TMDs in comparison to control populations, albeit without differentiating the analysis between chronic and acute/subacute forms [23, 24].

The CSS score was not significantly correlated with variation from baseline to follow-up in perceived quality of life, sleep quality, coping strategies or oral behavior in people with acute/subacute TMD. These results are in contrast to our initial expectation and could have been influenced by the relatively small sample size included. In fact, it is known that greater psychological distress is commonly associated with poorer sleep quality and less quality of life in people with TMDs [59]. With regards to the role of stress on oral behaviour, there is moderate evidence to indicate that higher levels of stress can lead to negative oral habits like parafunction [18]. Additionally, past studies examining patients with TMD found that high levels of catastrophizing and negative coping strategies were strongly associated with poorer psychosocial status, high psychological distress, anxiety, depression, pain intensity and disability [60–63].

In the current study, for those with chronic TMD, the variation in HADS from baseline to follow up was significantly correlated with the CSS score. This result indicates that for those with chronic TMD, their level of anxiety, depression and stress worsened due to the COVID-19 stress exposure. Previous research suggested that people with chronic TMD can present with high levels of anxiety and depression, and their mental state is significantly modified with stress exposure [41, 61, 64, 65]. Our results confirm this by revealing that the stress provoked by COVID-19 was associated with a significant increase in anxiety and depression in those with chronic TMD. By contrast, participants with acute/subacute TMD demonstrated no significant change on the HADS over the lockdown period.

The same finding was observed for variation of the CSI score and CSS for those with chronic TMD. The variation from baseline to follow for the CSI showed a significant positive correlation with CSS but only for those with chronic TMD. Central sensitization can have a relevant role in the onset and maintenance of pain in patients with chronic TMD [66, 67]. An observational study of people with TMD reported that emotional distress can influence TMD status and the correlation with central sensitization [68]. Our results reinforce these findings suggesting that the interaction between stress, central sensitization and chronic pain is complex, potentially constituting a causal chain.

The variation from baseline to follow up of the GCPS was also significantly correlated with CSS but only for those with chronic TMD. Several studies have revealed an association between stress, pain and disability in people with TMDs by identifying stress events as a possible amplifier of TMD symptoms [19, 28, 64, 65]. Even if no causal relation can be drawn, our prospective study provides new insight on the association between stress and chronic facial pain severity by showing that when patients with chronic TMD are exposed to stress, they experience greater pain and pain-related disability.

Our findings should be considered with caution given some limitations of the study. Firstly, the relatively small sample size with an unequal distribution of chronic and acute/subacute TMD participants may have influenced results. Secondly, this study was conducted in one Centre only in one Country, which reduces the external validity and the generalisability of the results. In addition, there are several other variables (e.g. fear-learning behaviours and measures of pain perception such as pressure pain threshold, thermal pain sensitivity, temporal summation) that were not measured but may have been relevant [69–71]. Moreover, no control group with asymptomatic participants was monitored. We used an Italian translation of the CSS since this measure has not yet been validated in Italian (see S1 Appendix). In our study, we also selected to consider the CSS in relation to the COVID-19 lockdown period (90 days, from March 2020 to the end of May 2020) and not to the last week as reported in the English version [53]. Furthermore, the CSS does not inquire about isolation-related stress that could have potentially impacted on symptoms during the COVID-19 lockdown. Another potential limitation is that we divided patients into acute/subacute TMD versus chronic TMD without considering the type of TMD diagnosis as a possible confounder. Finally, we did not consider other possible confounding factors during the lockdown period such as negative affective distress or loss of family or friends. Nevertheless, there are many strengths of this study. This is the first prospective study investigating the impact of COVID-19 related distress on people with TMDs. The prospective research methodology allowed us to examine the correlation between stress and the observed variables in addition to the correlation between stress and the variation of observed variables during the lockdown period. This methodological aspect is relevant as it allowed us to understand not only if people with TMD were affected by COVID-19 related stress (as previous studies have examined), but how they were affected [23, 24]. Thus, our study is the first prospective study to report how a global stressor can influence people with TMD in relation to their general health, oral behaviours, psychosocial features, disability and pain intensity. To know "How" and not only "If" stress affects TMD patients is fundamental from a clinical perspective to ensure appropriate management.

Another notable aspect of the current study is the analysis of both chronic and acute/subacute TMD. Our results showed that people with chronic TMD are more susceptible to COVID-19 related distress than those with acute/subacute symptoms. These data contribute to the understanding of the complex interaction between stress, psychological status and chronic pain in people with TMD.

## Conclusions

This study is the first prospective study describing how COVID-19 related distress affected people with TMD during lockdown in relation to their general health, oral behaviours, psychosocial features, disability and pain intensity. Our results revealed that people with chronic TMD were more susceptible to the distress caused by COVID-19 with a deterioration of their psychological status, worsening of CS and increased chronic facial pain severity. Although the generalisability of the results is limited, clinicians managing people with TMDs should consider these preliminary findings by providing greater support and care to these patients during the COVID-19 pandemic or other stressful events. The knowledge gained from this study contributes to a greater understanding of the role of psychological stress as a possible amplifier of CS, anxiety, depression, chronic pain and pain-related disability in people with TMDs.

## Supporting information

S1 Appendix CSS Italian translation. Scale di misura dello stress da COVID-19 (Original Version: Taylor et al., 2020).
(DOCX)
