# Supplementary material for: The impact of Covid-19-related distress on general health, oral behaviour, psychosocial features, disability and pain intensity in a cohort of Italian patients with temporomandibular disorders
Source: PLoS One. 2021 Feb 2;16(2):e0245999. doi: 10.1371/journal.pone.0245999 (PMC7853459; doi:10.1371/journal.pone.0245999)
Supplement: S1 Appendix — Scale di misura dello stress da COVID-19 (Original Version: Taylor et al., 2020). (DOCX) [file pone.0245999.s001.docx]

| **Di seguito vengono indicati i vari tipi di preoccupazioni che potrebbe aver provato durante il lockdown degli ultimi 3 mesi.**  **Nelle seguenti dichiarazioni ci riferiamo a COVID-19 come "il virus".** | | 0 | 1 | 2 | 3 | 4 |
| --- | --- | --- | --- | --- | --- | --- |
|  |  | Per niente | Leggermente | Moderatamente | Molto | Estremamente |
| P | Sono preoccupato di prendere il virus |  |  |  |  |  |
| P | Sono preoccupato che l'igiene di base (ad esempio il lavaggio delle mani) non sia sufficiente per proteggermi dal virus |  |  |  |  |  |
| P | Sono preoccupato che il nostro sistema sanitario non sia in grado di proteggermi dal virus |  |  |  |  |  |
| P | Sono preoccupato di non poter proteggere la mia famiglia dal virus |  |  |  |  |  |
| P | Sono preoccupato che il nostro sistema sanitario non sarà in grado di proteggere i miei cari |  |  |  |  |  |
| P | Sono preoccupato che il distanziamento sociale non sia sufficiente per proteggermi dal virus |  |  |  |  |  |
| CSE | Sono preoccupato che i negozi di alimentari restino a corto di cibo |  |  |  |  |  |
| CSE | Sono preoccupato che i negozi di alimentari restino sprovvisti di rimedi per il raffreddore o l'influenza |  |  |  |  |  |
| CSE | Sono preoccupato che le farmacie restino sprovvisti di farmaci da banco |  |  |  |  |  |
| CSE | Sono preoccupato che i negozi di alimentari restino sprovvisti di acqua |  |  |  |  |  |
| CSE | Sono preoccupato che i negozi di alimentari restino sprovvisti di prodotti per la pulizia o disinfettanti |  |  |  |  |  |
| CSE | Sono preoccupato che i negozi di alimentari chiudano |  |  |  |  |  |
| X | Sono preoccupato che gli stranieri stiano diffondendo il virus nel mio paese |  |  |  |  |  |
| X | Se incontrassi una persona di un paese straniero sarei preoccupato che possa avere il virus |  |  |  |  |  |
| X | Sono preoccupato di entrare in contatto con stranieri perché potrebbero avere il virus |  |  |  |  |  |
| X | Sono preoccupato che gli stranieri stiano diffondendo il virus perché non sono così puliti come noi |  |  |  |  |  |
| X | Se andassi in un ristorante specializzato in cibi stranieri sarei preoccupato di prendere il virus |  |  |  |  |  |
| X | Se fossi in un ascensore con un gruppo di stranieri, sarei preoccupato che siano infetti dal virus |  |  |  |  |  |
| C | Sono preoccupato che le persone intorno a me mi infettino con il virus |  |  |  |  |  |
| C | Sono preoccupato che se toccassi qualcosa in uno spazio pubblico (ad esempio corrimano, maniglia della porta) potrei prenderei il virus |  |  |  |  |  |
| C | Sono preoccupato che se qualcuno tossisse o starnutisse vicino a me potrei prendere il virus |  |  |  |  |  |
| C | Sono preoccupato di prendere il virus dal maneggiare denaro o dall'utilizzo di un bancomat |  |  |  |  |  |
| C | Sono preoccupato dal prendere il resto nei pagamenti in contanti |  |  |  |  |  |
| C | Sono preoccupato che la mia posta sia stata contaminata dagli smistatori della posta |  |  |  |  |  |

| **Nelle seguenti dichiarazioni ci riferiamo a COVID-19 come "il virus".**  **Per favore legga ogni affermazione e indichi la frequenza con cui si è verificato ciascun problema durante il lockdown degli ultimi 3 mesi.** | | 0 | 1 | 2 | 3 | 4 |
| --- | --- | --- | --- | --- | --- | --- |
|  |  | Mai | Raramente | Qualche volta | Spesso | Quasi sempre |
| ST | Ho avuto problemi a dormire perché ero preoccupato per il virus |  |  |  |  |  |
| ST | Ho fatto brutti sogni sul virus |  |  |  |  |  |
| ST | Ho pensato al virus quando non volevo |  |  |  |  |  |
| ST | Immagini mentali inquietanti sul virus mi sono venute in mente contro la mia volontà |  |  |  |  |  |
| ST | Ho avuto problemi di concentrazione perché continuavo a pensare al virus |  |  |  |  |  |
| ST | Ricordarsi del virus mi ha causato reazioni fisiche, come sudorazione o battiti accelerati |  |  |  |  |  |

| **Le seguenti voci riguardano informazioni sui comportamenti.**  **durante il lockdown degli ultimi 3 mesi quanto spesso ha avuto uno di questi comportamenti a causa delle preoccupazioni su COVID-19?** | | 0 | 1 | 2 | 3 | 4 |
| --- | --- | --- | --- | --- | --- | --- |
|  |  | Mai | Raramente | Qualche volta | Spesso | Quasi sempre |
| R | Cercato post sui social media riguardanti COVID-19 |  |  |  |  |  |
| R | Cercato video di YouTube su COVID-19 |  |  |  |  |  |
| R | Cercato rassicurazione da amici o parenti su COVID-19 |  |  |  |  |  |
| R | Controllato il proprio corpo per segni di infezione (ad esempio misurazione della temperatura) |  |  |  |  |  |
| R | Chiesto consigli a professionisti sanitari (ad esempio medici o farmacisti) su COVID-19 |  |  |  |  |  |
| R | Cercato in Internet trattamenti per COVID-19 |  |  |  |  |  |
